# B-Vitamin Intake from Diet and Supplements and Breast Cancer Risk in Middle-Aged Women: Results from the Prospective NutriNet-Santé Cohort

**DOI:** 10.3390/nu9050488

**Published:** 2017-05-13

**Authors:** Manon Egnell, Philippine Fassier, Lucie Lécuyer, Laurent Zelek, Marie-Paule Vasson, Serge Hercberg, Paule Latino-Martel, Pilar Galan, Mélanie Deschasaux, Mathilde Touvier

**Affiliations:** 1 Sorbonne Paris Cité Epidemiology and Statistics Research Center (CRESS), Inserm U1153, Inra U1125, Cnam, Paris 13 University, Nutritional Epidemiology Research Team (EREN), 74 rue Marcel Cachin, 93017 Bobigny, France; manon.egnell@agroparistech.fr (M.E.); l.lecuyer@eren.smbh.univ-paris13.fr (L.L.); laurent.zelek@avc.aphp.fr (L.Z.); s.hercberg@eren.smbh.univ-paris13.fr (S.H.); paule.martel@jouy.inra.fr (P.L.-M.); p.galan@eren.smbh.univ-paris13.fr (P.G.); m.deschasaux@eren.smbh.univ-paris13.fr (M.D.); m.touvier@eren.smbh.univ-paris13.fr (M.T.); 2 French Network for Nutrition and Cancer Research (NACRe Network), 78352 Jouy-en-Josas, France; m-paule.vasson@udamail.fr; 3 Oncology Department, Avicenne Hospital, 93017 Bobigny, France; 4 UFR Pharmacie, Inra, UMR 1019, CRNH Auvergne, Centre Jean-Perrin, CHU Gabriel-Montpied, Unité de Nutrition, Clermont Université, Université d’Auvergne, 63000 Clermont-Ferrand, France; 5 Public Health Department, Avicenne Hospital, 93017 Bobigny, France

**Keywords:** B-vitamins, diet, dietary supplements, prospective cohort, breast cancer risk

## Abstract

Experimental studies suggest a protective effect of B-vitamins on breast cancer risk, potentially modulated by alcohol intake. However, epidemiological studies are limited, especially regarding non-folate B-vitamins. Furthermore, few studies included quantitative assessment of supplemental intake. This prospective study aimed to investigate the associations between intakes of B-vitamins (dietary, supplemental, total) and breast cancer risk. 27,853 women aged ≥45 years from the NutriNet-Santé cohort (2009–2016) were included, with a median follow-up time of 4.2 years. Dietary data were collected using repeated 24 h records. A specific questionnaire assessed dietary supplement use over a 12-month period. A composition database of 8000 supplements was developed. Associations were characterized by multivariable Cox models, and 462 incident breast cancers were diagnosed. Dietary (HR_Q4vs.Q1_ = 0.74 (0.55, 0.99), *P*-trend = 0.05), supplemental (HR_Q4vs.Q1_ = 0.61 (0.38, 0.98), *P*-trend = 0.05), and total (HR_Q4vs.Q1_ = 0.67 (0.50, 0.91), *P*-trend = 0.01) pyridoxine intakes were inversely associated with breast cancer risk. Total thiamin intake was borderline inversely associated with breast cancer risk (HR_per 1-unit increment_ = 0.78 (0.61, 1.00), *P* = 0.05). Statistically significant interactions between alcohol consumption and B-vitamin (thiamin, riboflavin, niacin, pantothenic acid, pyridoxine, folate, and cobalamin) supplemental intake were observed, the latter being inversely associated with breast cancer risk in non-to-low alcohol drinkers but not in higher drinkers. This large prospective study, including quantitative assessment of supplemental intake, suggests a potential protective effect of pyridoxine and thiamin on breast cancer risk in middle-aged women.

## 1. Introduction

Breast cancer is the most common cancer among women in the world, with approximatively 1.67 million new cases of breast cancer diagnosed in 2012; and nearly 522,000 associated deaths [1]. Experimental studies have suggested protective effects of micronutrients involved in one-carbon metabolism—such as B-vitamins (folate, thiamine, riboflavin, niacin, pantothenic acid, pyridoxine, cobalamin)—on breast cancer risk, notably through genetic and epigenetic mechanisms. One carbon-metabolism is a network of biochemical pathways that provides methyl groups. Its disruption can promote carcinogenesis by interfering with DNA replication, DNA repair, or the regulation of gene expression [2].

Folate has been by far the most investigated B-vitamin. A limited number of placebo-controlled randomized trials have evaluated the effect of folic acid supplementation on breast cancer risk. In 2013, a meta-analysis of such studies showed null results (13 included trials) [3]. Few randomized controlled trials have been conducted to test the effect of other B-vitamins but cancer was not the primary outcome, and no significant effect on breast cancer risk was observed [4]. Even if randomized trials can provide evidence regarding the causality of the studied associations, observational studies provide complementary information since they are based on real exposures in terms of nutrient associations, sources (dietary, supplemental), and doses.

In 2010, the World Cancer Research Fund and the American Institute for Cancer Research (WCRF/AICR) conducted a systematic literature review [5] that included twelve prospective observational studies on breast cancer risk and folate intake, two on cobalamin, only one for riboflavin, one for pyridoxine, and two on B-vitamin supplement use. For all B-vitamins, the level of proof was judged “limited-non conclusion” [5]. Since then, few epidemiological prospective studies investigated the associations between intakes of B-vitamins and breast cancer risk. Most of them focused on folate intakes [6,7,8,9,10,11,12,13,14] and showed contrasting results. Even if some studies suggested an inverse association between folate intakes and breast cancer risk [6,7,11,12], most of the others did not show any significant association [7,9,10,12,14]. For other B-vitamins, the literature is scarcer [4,9] and only one prospective cohort study found an inverse significant association between dietary intakes of thiamin, riboflavin, and pyridoxine and breast cancer risk [6]. Moreover, a limited number of prospective studies specifically investigated the associations between B-vitamin supplement use and breast cancer risk [8,11,15,16,17,18,19,20] and even less took into account supplement doses [8,11,15,16,17]. Dietary supplement use is constantly increasing in western countries and their omission in many European epidemiological studies may lead to underestimating the intake [21]. Besides, a given nutrient may have differential effects according to its vector (food or supplement).

Furthermore, experimental evidence suggested a potential interaction between B-vitamins and alcohol intake (especially folates) by interfering with their transport, absorption, and metabolism, which may modulate the association with breast cancer risk [22]. However results of the prospective epidemiological studies that investigated such interaction mostly focused on folates and showed contrasted results [6,9,10,11,14,15,16,17,18,19,23,24,25]. Some of them suggested that low folate intake could be associated with an increased breast cancer risk among women with higher alcohol consumption ([16,19]) while many did not show any significant association [6,9,10,11,14,15,17,18,23,24,25]. Few prospective studies examined the interactions between alcohol intake and other B-vitamins [6,9,11,24,25]. 

Thus, this study aimed at investigating the associations between intakes of B-vitamins (dietary, supplemental, and total) and breast cancer risk in middle-aged women, and their potential modulation by alcohol intake. 

## 2. Material and Methods

### 2.1. The NutriNet-Santé Cohort

The NutriNet-Santé study is a French ongoing web-based cohort launched in 2009 with the objective of studying the associations between nutrition and health as well as the determinants of dietary behaviors and nutritional status. This cohort has been previously described in detail [26]. Participants aged over 18 years with access to the Internet are recruited by vast multimedia campaigns. All questionnaires are completed online using a dedicated website (https://www.etude-nutrinet-sante.fr/). The NutriNet-Santé study is conducted according to the Declaration of Helsinki guidelines. It was approved by the Institutional Review Board of the French Institute for Health and Medical Research (IRB Inserm n°0000388FWA00005831) and the “Commission Nationale de l’Informatique et des Libertés” (CNIL n°908450/n°909216). Electronic informed consent is obtained from each participant (EudraCT No. 2013-000929-31).

### 2.2. Data Collection

At inclusion, participants completed a set of five questionnaires related to socio-demographic and lifestyle characteristics [27] (e.g., occupation, educational level, smoking status, alcohol consumption, number of children), anthropometrics [28,29] (e.g., height, weight), dietary intakes (see below), physical activity (validated IPAQ questionnaire) [30], and health status (e.g., personal and family history of diseases, medication use—including hormonal treatment for menopause—and menopausal status). Age at menopause was collected in the yearly health status questionnaires.

#### 2.2.1. Dietary Data

Usual dietary intakes were assessed every six months through a series of three non-consecutive validated web-based 24 h-dietary records, randomly assigned over a 2-week period (2 weekdays and 1 weekend day) [31,32,33]. Mean dietary intake during the first year of each participant’s follow-up was considered as baseline dietary intake in this prospective analysis. Participants used a dedicated interface of the study website to declare all foods and beverages consumed during a 24 h-period: three main meals (breakfast, lunch, dinner) or any other eating occasion. Portion sizes were estimated using validated photographs [34]. Mean daily energy, alcohol, and nutrient intakes were estimated using a published French food composition table (>3300 items) [35]. Amounts consumed from composite dishes were estimated using French recipes validated by food and nutrition professionals. Dietary underreporting was identified on the basis of the method proposed by Black, using the basal metabolic rate and Goldberg cut-off, and under-energy reporters were excluded [36]. The present study focused on B-vitamins (thiamin, riboflavin, niacin, pantothenic acid, pyridoxine, folate, and cobalamin). 

#### 2.2.2. Dietary Supplement Use

Two months after their inclusion, participants were invited to fill a questionnaire regarding dietary supplement use [37]. In the questionnaire, participants were asked whether they took any supplement during the past 12 months (at least once). In this open-ended questionnaire, they had to specify the name and the brand of the dietary supplement, the form used, the number of days of consumption, and the number of units generally used when they consumed the supplement. All dietary supplement compositions have been collected from brand websites or DS packagings, creating a database with quantitative content information for about 8000 products. Then, the average daily intake of dietary supplements during the last 12 months has been assessed for each subject, using this composition database. In the present study, true dietary supplements and medicinal supplements (supplements considered as pharmaceutical products in France, and mainly composed of vitamins and minerals) were both considered as dietary supplements. However, for each nutrient, participants whose supplemental intake exceeded the 99th percentile by using a medicinal supplement were excluded.

### 2.3. Case Ascertainment 

Participants self-declared health events through the yearly health status questionnaire, through a specific check-up questionnaire for health events (every three months) or at any time through an interface on the study website. Following this declaration, participants were invited to send their medical records (diagnosis, hospitalization, etc.) and, if necessary, the study physicians contacted the participants’ treating physician or the medical structures to collect additional information. Then, data are reviewed by an independent physician expert committee for the validation of major health events. Cancer cases were classified using the International Chronic Diseases Classification, 10th Revision, Clinical Modification (ICD-10) [38]. In this study, all first primary breast cancers diagnosed between the inclusion and April 2016 were considered as cases.

### 2.4. Statistical Analyses 

So far, 57,703 women provided at least three valid 24 h-dietary records during the first year of follow-up and answered the dietary supplements questionnaire. Women aged under 45 years were excluded (*n* = 29,850) because of a very low susceptibility to developing breast cancer. Thus, 27,853 middle-aged women were included in the analyses. For all covariates, there was no missing data except for physical activity (12.7%) and educational level (5%), for which multiple imputation was performed [39]. B-vitamin dietary intake was introduced into the models as continuous variables and quartiles. Supplemental intake was coded as users/non users, and as a four-category variable: non-users and tertiles of supplemental intake in users. Total (dietary + supplemental) intake was introduced into the models as continuous variables and quartiles.

Associations between dietary, supplemental, and total intake for each B-vitamin and breast cancer risk were characterized (HR and 95% CI) using multivariable Cox proportional hazard models with age as the primary time variable. Women contributed person-time to the Cox model until the date of cancer diagnosis, the date of the last completed questionnaire, the date of death or April 2016, whichever occurred first. 

Models were adjusted for age (time-scale), BMI (kg/m^2^, continuous), height (cm, continuous), physical activity (high, moderate, low, computed following IPAQ recommendations [31]), smoking status (never smokers, former smokers, smokers), number of dietary records (continuous), alcohol intake (g/day, continuous), energy intake (without alcohol, g/day, continuous), family history of cancer (yes/no), educational level (<high-school degree, <2 years after high-school degree, ≥2 years after high-school degree), number of biological children (continuous), menopausal status at baseline (pre-menopause, perimenopause, post-menopause), and hormonal treatment for menopause (yes/no). Tests for linear trends were performed across categories with the use of the ordinal value of the corresponding variable. 

Interactions between dietary, supplemental, and total intake of each B-vitamin and the following potential modifiers were tested by introducing the product of the two variables into Cox models performed on the whole population study: smoking status (current smokers/non- or former smokers), alcohol intake (<3.6 g/day/≥3.6 g/day, i.e., the population median), and family history of cancer (yes/no). Stratified analyses were performed when p-values for interaction were statistically significant. Analyses were also performed according to menopausal status: women contributed person-time to the “pre-menopause model” until their age of menopause and to the “post-menopause model” from their age of menopause. Models restricted to the ER+ breast cancer subtype were also performed. Statistical power was too limited to perform separate models for the ER− breast cancer subtype. Since the association between baseline dietary exposure and cancer risk may decrease as the follow-up time increases [40], we have carried out separate analyses during the first half and the second half of the follow-up period.

A *p*-value ≤ 0.05 was considered statistically significant. All analyses were carried out with SAS software (version 9.4; SAS Institute, Inc., Cary, NC, USA). 

## 3. Results

Between May 2009 and April 2016 (median follow-up time: 4.2 years; 111,200 person-years), 462 incident breast cancer cases were diagnosed: 78 premenopausal and 384 postmenopausal; 72.6% ER+/PR+, 12.4% ER−/PR−, 14.6% ER+/PR−, 0.4% ER−/PR+; 82.2% invasive and 17.8% in situ. The mean age at diagnosis was 59.8 years (SD = 7.2) and mean baseline-to-diagnosis time was 2.5 years (SD = 1.7). The mean number of dietary records per subject over their first year of follow-up was 4.8 (SD = 1.6). The baseline characteristics of participants are described in Table 1. Cancer risk was higher in women who were taller, who had family history of cancer, higher alcohol intake, and a medium level of education.

Table 2 displays the association between dietary, supplemental, and total intake of the different B vitamins (thiamin, riboflavin, niacin, pantothenic acid, pyridoxine, folate, and cobalamin) and breast cancer risk. Dietary (HR_Q4vs.Q1_ = 0.74 (0.55, 0.99), *P*-trend = 0.05), supplemental (HR_Q4vs.Q1_ = 0.61 (0.38, 0.98), *P*-trend = 0.05), and total (HR_Q4vs.Q1_ = 0.67 (0.50, 0.91), *P*-trend = 0.01) pyridoxine intakes were inversely associated with breast cancer risk. Total thiamin intake was inversely associated with breast cancer risk (HR_per 1-unit increment_ = 0.78 (0.61, 1.00), *P* = 0.05). No association was observed between the other B-vitamins and breast cancer risk. 

Statistically significant interactions between alcohol consumption and supplemental intakes of B-vitamins were observed (*P* = 0.003 for thiamin, *P* = 0.007 for riboflavin, *P* = 0.01 for niacin, *P* = 0.01 for pantothenic acid, *P* = 0.02 for pyridoxine, *P* = 0.02 for folate, and *P* = 0.02 for cobalamin, Table 3). In stratified analyses, supplemental intakes of B-vitamins were associated with a decreased breast cancer risk among women with non-to-low alcohol consumption (HR_users vs.non-users_ = 0.52 (0.30, 0.87), *P* = 0.01 for thiamin; HR_users vs.non-users_ = 0.55 (0.33, 0.93), *P* = 0.02 for riboflavin; HR_users vs.non-users_ = 0.53 (0.31, 0.90), *P* = 0.02 for niacin; HR_users vs.non-users_ = 0.47 (0.26, 0.84), *P* = 0.01 for pantothenic acid; HR_users vs.non-users_ = 0.59 (0.39, 0.89), *P* = 0.01 for pyridoxine; HR_users vs.non-users_ = 0.60 (0.37, 0.98), *P* = 0.04 for folate; and HR_users vs.non-users_ = 0.50 (0.26, 0.97), *P* = 0.04 for cobalamin), while none of these associations were statistically significant in women with alcohol intake above the population median. No interaction was detected between alcohol intake and dietary or total intake of B-vitamins, nor with smoking status or family history of cancer with any type of intake (all *p* > 0.05, data not tabulated).

Compared to the whole population, results of the associations between pyridoxine and thiamine intakes and breast cancer risk tended to be similar when models focused on postmenopausal women (*n* = 384 cases, HR_Q4vs.Q1_ = 0.60 (0.43, 0.84), *P*-trend = 0.005 for dietary, HR_Q4vs.Q1_ = 0.54 (0.31, 0.94), *P*-trend = 0.06 for supplemental, and HR_Q4vs.Q1_ = 0.70 (0.50, 0.98), *P*-trend = 0.04 for total intake of pyridoxine, and HR_per 1-unit increment_ = 0.82 (0.63, 1.05), *P* = 0.1 for total intake of thiamine). Statistical power was very limited in premenopausal women (*n* = 118 cases) and no statistically significant association was detected (corresponding *p*-values = 0.08, 0.7, 0.2, and 0.2, respectively) (data not tabulated). Results of the associations between the different types of pyridoxine intake and breast cancer risk tended to be stronger in the first half of the follow-up period (first 3.5 years) (HR_Q4vs.Q1_ = 0.72 (0.54, 0.97), *P*-trend = 0.03) for dietary intake, HR_Q4vs.Q1_ = 0.61 (0.38, 0.98), *P*-trend = 0.06 for supplemental intake and HR_Q4vs.Q1_ = 0.66 (0.49, 0.89), *P*-trend = 0.01 for total intake) compared to the second half of follow-up (HR_Q4vs.Q1_ = 0.76 (0.44, 1.31), *P*-trend = 0.3) for dietary intake, HR_Q4vs.Q1_ = 0.25 (0.06, 1.00), *P*-trend = 0.2 for supplemental intake and HR_Q4vs.Q1_ = 0.51 (0.28, 0.92), *P*-trend = 0.06 for total intake). However, results of the associations between thiamine intakes and breast cancer risk were similar between the two halves of the follow-up period. Analyses restricted to the ER+ breast cancer subtype (*n* = 426) have been carried out, however no significant association was observed. Regarding the ER− breast cancer subtype (*n* = 36), a lack of power did not allow us to carry out the analyses among this group. Furthermore, similar trends, although less pronounced, were observed when Cox models were only age-adjusted (Supplemental Table S1). Results were very similar when missing values for covariates (education level and physical activity) were replaced by the modal category instead of multiple imputation. Similar trends for all results were observed when analyses excluded cases diagnosed during their first year of follow-up (361 cases/27,853 non-cases included) or when analyses were restricted to invasive breast cancers (295 cases/27,853 non-cases) (data not shown). 

## 4. Discussion

In this large prospective cohort study, dietary, supplemental, and total pyridoxine intake and total thiamin intake were associated with decreased breast cancer risk. No significant association between other intakes of B-vitamins and breast cancer risk was found overall. A modulation by alcohol intake was suggested by statistically significant interactions: the use of thiamin, riboflavin, niacin, pantothenic acid, folate, and cobalamin supplements was associated with lower breast cancer risk in non-to-low alcohol drinkers, while no association was observed in women with higher alcohol intake. However, no interaction was observed between other types of B-vitamin intakes and alcohol intake, or between any type of B-vitamin intake and smoking status or family history of cancer.

While a substantial body of literature is available regarding folate and breast cancer risk [3,5], pyridoxine has been less studied and the results are contrasted. To our knowledge, few randomized trials investigated the effect of a supplementation with pyridoxine (combined or not with cobalamin and folates) on breast cancer risk, but none were designed with breast cancer as the primary outcome, and all obtained null results [4]. As recently reviewed and meta-analysed [4], few prospective observational studies have investigated this relationship. While several prospective studies observed no association between dietary or total pyridoxine intake and breast cancer risk, a prospective cohort study on 10,786 Italian women from the ORDET (Hormones and Diet in the Etiology of Breast Cancer Risk) cohort found a decreased breast cancer risk associated with dietary intakes of pyridoxine [6]. Consistently, some cohort studies which investigated the association between breast cancer risk and plasma pyridoxine concentration have observed a significant inverse relationship [4,41,42]. Pyridoxine is involved in one-carbon metabolism as an enzyme cofactor for cystathionine β-synthase and cystathionine γ-lyase [43]. It may also protect against breast cancer though mechanisms independent of one-carbon metabolism. Pyridoxine plays a role in the inflammatory response, which is supposed to be involved in the development and progression of breast cancer [44], through the production of inflammation-mediating cytokines [45] and the activation of lymphocytes [46]. 

The epidemiological literature is even scarcer regarding the association between thiamin intake and breast cancer risk. A prospective study including 49,654 Canadian women observed no association between dietary thiamin intake and breast cancer risk [9], while higher dietary thiamin intake was associated with decreased breast cancer risk in a recent prospective study on women from the ORDET cohort [6], consistent with our results. Thiamin is an enzyme cofactor involved in metabolic processes that is often altered in tumor tissue and may play a role in DNA synthesis [47].

In our study, no overall association was observed between dietary, supplemental, or total folate intake and breast cancer risk, which may partly be explained by the relatively low intakes. This result is consistent with those of a meta-analysis of randomized trials on folic acid supplementation [3], with the conclusions of the WCRF/AICR 2010 CUP report [5], and with two recent meta-analyses of observational studies, including respectively 12 cohorts and 12 nested case-control studies for one and 20 cohort studies for the other [14,48]. However, the mechanistic hypothesis supports a potential role of folate in breast cancer aetiology. Indeed, folate is involved in DNA and RNA methylation and in different key mechanisms such as the conversion of homocysteine to methionine and the production of S-adenosyl methionine. Low folate intake could alter DNA methylation and then affect DNA integrity and stability [7]. The fact that epidemiological findings fail to confirm the mechanistic hypothesis from the experimental data suggests that other individual factors may interact with folate and other intakes of B-vitamins and makes the global picture more complex.

For instance, an interaction between B-vitamins, and especially folate intake, and alcohol intake has been hypothesized [6,9,10,11,14,15,16,17,18,19,23,24,25]. In our study, alcohol intakes were relatively low, which could explain that we did not observe interactions between dietary folate or other B-vitamins and alcohol intakes. However, we observed a statistically significant interaction between each B-vitamin from dietary supplements and alcohol intake. Some studies have suggested that low folate intakes could be associated with an increased breast cancer risk among women with higher alcohol consumption [16,19]. In our study, we found an inverse association between B-vitamin supplements and breast cancer risk only for women with non-to-low alcohol intakes. This result has been also observed in the case-control study of Yang et al., who showed a decreased risk of ER− breast cancer among women who reported a low alcohol intake and higher folate intake [49]. In a recent prospective study, Cancarini et al. also found that folic acid intake was associated with reduced breast cancer risk in non-to-low alcohol drinkers [6]. Concerning the other B-vitamins, one study tested the interactions between alcohol and thiamin, riboflavin, and niacin on breast cancer risk, but no significant interaction was observed [9]. Alcohol and acetaldehyde, its primary metabolite which is mutagenic and carcinogenic, are known to be antagonists of folate, limiting its absorption and metabolism, and interfering with DNA methylation and repair [50]. 

To our knowledge, very few studies investigated the interactions between smoking status or family history of cancer and intake of B-vitamins on breast cancer risk [51,52]. In our study we did not observe any significant interaction with smoking status and family history of cancer. Regarding the literature, a case-control study has found an inverse association between dietary folate and breast cancer risk among non-smokers, while no significant association was found among smokers [51]. 

The strengths of our study pertained to its prospective design, its large sample size, and the quantitative assessment of nutritional intake from dietary supplements. Nevertheless, some limitations should be acknowledged. First, caution is needed regarding the extrapolation of these results to the entire French population since this study included volunteers involved in a long-term cohort study investigating the association between nutrition and health, with overall more health-conscious behaviors and higher socio-professional and educational levels. Thus, unhealthy dietary behaviors may have been underrepresented in this study, which may have weakened the observed associations. Next, regarding dietary supplement intake, the questionnaire only covered 12 months at the beginning of the follow-up. An update of these data later during follow-up would improve the precision of the exposure assessment. Moreover, despite the careful collection of data on dietary intake and supplement use, the lack of biomarker status measurement may have led to the misclassification of some participants [53]. Finally, residual confounding cannot be ruled out. However, analyses were adjusted for a wide range of cofactors, thereby limiting this potential bias.

In conclusion, this prospective cohort study suggests that pyridoxine and thiamin could be inversely associated with breast cancer risk, in line with mechanistic hypotheses. Alcohol intake may modulate the associations between B-vitamin supplement use and breast cancer risk. These results need to be confirmed in future large prospective observational and interventional studies before public health recommendations could be derived. In the meantime, reaching an adequate intake of B-vitamins through a balanced diet should be recommended, rather than regular use of dietary supplements, of which the long term effects still need deeper investigation.

## Figures and Tables

**Table 1:** Baseline characteristics of the study population (*n* = 27,853), NutriNet-Santé Cohort, France, 2009–2016.

	Overall		Cases (*n* = 462)		Non-Cases (*n* = 27,391)		*p* ^a^
	*N*%	Mean ± SD	*N* (%)	Mean ± SD	*N* (%)	Mean ± SD	
Age, years		60.2 ± 7.3		59.8 ± 7.2		60.2 ± 7.3	0.2
Educational level							0.006
<high-school degree	7911 (28.4)		109 (23.6)		7802 (28.5)		
≥high-school degree to <2 years after high-school degree	4941 (17.7)		105 (22.7)		4836 (17.7)		
≥2 years after high-school degree	15,001 (53.9)		248 (53.7)		14,753 (53.8)		
Smoking status							0.2
Non-smokers	13,441 (48.3)		227 (49.1)		13,214 (48.2)		
Former smokers	11,318 (40.6)		196 (42.4)		11,122 (40.6)		
Smokers	3094 (11.1)		39 (8.5)		3055 (11.2)		
Physical activity ^b^							0.7
Low	4975 (17.9)		79 (17.1)		4896 (17.9)		
Moderate	10,007 (35.9)		161 (34.8)		9846 (35.9)		
High	9346 (33.6)		167 (36.1)		9179 (33.5)		
BMI, kg/m^2^		24.3 ± 4.7		24.5 ± 4.6		24.3 ± 4.7	0.4
Height, cm		163 ± 6		164 ± 6		163 ± 6	0.003
Energy intake without alcohol, kcal/day		1700 ± 380		1700 ± 354		1700 ± 380	0.8
Alcohol intake, g/day		6.9 ± 9.4		8.0 ± 9.9		6.9 ± 9.4	0.02
Alcohol intake among drinkers, g/day	19,912 (71.5)	9.7 ± 9.8	361 (78.1)	10.3 ± 10.2	19,551 (71.4)	9.7 ± 9.8	0.3
Number of biological children		1.9 ± 1.1		2.0 ± .1		1.9 ± 1.1	
Family history of cancer ^c^ (yes)	14,389 (51.7)		270 (58.4)		14,119 (51.5)		0.003
Menopausal status							0.4
Pre-menopausal	7688 (27.6)		119 (25.8)		7569 (27.6)		
Post-menopausal	20,165 (72.4)		343 (74.2)		19,822 (72.4)		
Use of hormonal treatment for menopause (yes) ^d^	3595 (12.9)		73 (15.8)		3522 (12.9)		0.06
Dietary intake of thiamin, mg/day		1.1 ± 0.4		1.1 ± 0.4		1.1 ± 0.4	0.4
Supplemental intake of thiamin, mg/day ^e^	3421 (12.3)		56 (12.1)	0.2 ± 0.4	3365 (12.3)	0.7 ± 5.6	<0.0001
Total intake of thiamin, mg/day		1.2 ± 2.0		1.1 ± 0.4		1.2 ± 2.0	0.0005
Dietary intake of riboflavin, mg/day		1.7 ± 0.5		1.7 ± 0.5		1.7 ± 0.5	0.4
Supplemental intake of riboflavin, mg/day ^e^	3449 (12.4)		56 (12.1)	0.2 ± 0.4	3393 (12.4)	0.5 ± 1.8	<0.0001
Total intake of riboflavin, mg/day		1.8 ± 0.8		1.8 ± 0.5		1.8 ± 0.8	0.5
Dietary intake of niacin, mg/day		18.1 ± 5.6		18.2 ± 5.5		18.1 ± 5.6	0.6
Supplemental intake of niacin, mg/day ^e^	3397 (12.2)		52 (11.3)	3.0 ± 4.7	3345 (12.2)	4.2 ± 18.9	0.1
Total intake of niacin, mg/day		18.6 ± 8.8		18.6 ± 5.8		18.6 ± 8.8	0.9
Dietary intake of pantothenic acid, mg/day		5.2 ± 1.4		5.2 ± 1.3		5.2 ± 1.4	0.7
Supplemental intake of pantothenic acid, mg/day ^e^	3160 (11.3)		46 (10.0)	1.1 ± 1.4	3114 (11.4)	2.6 ± 10.9	<0.0001
Total intake of pantothenic acid, mg/day		5.5 ± 4.0		5.3 ± 1.4		5.5 ± 4.0	0.02
Dietary intake of pyridoxine, mg/day		1.7 ± 0.5		1.7 ± 0.5		1.7 ± 0.5	0.5
Supplemental intake of pyridoxine, mg/day ^e^	5165 (18.5)		76 (16.5)	0.6 ± 0.9	5089 (18.6)	2.1 ± 9.0	<0.0001
Total intake of pyridoxine, mg/day		2.1 ± 4.0		1.8 ± 0.6		2.1 ± 4.0	<0.0001
Dietary intake of folate, µg/day		336 ± 106		337 ± 98		336 ± 106	0.8
Supplemental intake of folate, µg/day ^e^	3573 (12.8)		56 (12.1)	50.3 ± 101.9	3517 (12.8)	96.7 ± 1674.6	0.1
Total intake of folate, µg/day		348 ± 607		343 ± 106		348 ± 612	0.4
Dietary intake of cobalamin, µg/day		5.4 ± 5.0		5.7 ± 5.4		5.4 ± 5.0	0.2
Supplemental intake of cobalamin, µg/day ^e^	2239 (8.0)		35 (7.6)	0.9 ± 3.7	2204 (8.0)	4.7 ± 34.8	<0.0001
Total intake of cobalamin, µg/day		5.8 ± 11.1		5.8 ± 5.5		5.8 ± 11.2	1.0

SD, Standard Deviation; BMI, Body Mass Index ^a^ *p*-values based on the chi-square test for categorical variables and Student *T*-test for continuous variables; ^b^ From the validated IPAQ (International Physical Activity Questionnaire) questionnaire. Data available for 24,328 women; ^c^ In first degree relatives; ^d^ Among menopausal women; ^e^ Number (%) of users of the corresponding supplement, and mean daily intake (SD) among users only.

**Table 2:** Associations between dietary, supplemental, and total B vitamin intake and breast cancer risk, from multivariable Cox proportional hazards models ^a^, NutriNet-Santé Cohort, France, 2009–2016.

	Dietary Intake					Supplemental Intake						Total Intake			
		Cases/Non Cases	HR	95% CI	*P*-Trend		Cases/Non Cases	HR	95% CI	*P*-Trend		Cases/Non Cases	HR	95% CI	*P*-Trend
Thiamin	Continuous	462/27,376	0.85	[0.64–1.14]	0.3	Users vs. non-users	462/27,376	0.94	[0.71–1.24]	0.7	Continuous	462/27,376	0.78	[0.61–1.00]	0.05
*Vitamin B1*	Q1 ^b^	121/6820	1	(ref)	0.1	C1	406/24,026	1	(ref)	0.2	Q1	120/6817	1	(ref)	0.1
	Q2	113/6857	0.84	[0.64–1.09]		C2	26/1125	1.31	[0.88–1.95]		Q2	110/6853	0.82	[0.63–1.07]	
	Q3	125/6848	0.90	[0.69–1.18]		C3	18/1099	0.89	[0.56–1.43]		Q3	134/6844	0.98	[0.75–1.27]	
	Q4	103/6851	0.76	[0.56–1.02]		C4	12/1126	0.61	[0.34–1.08]		Q4	98/6862	0.72	[0.53–0.96]	
Riboflavin	Continuous	462/27,383	1.01	[0.83–1.24]	0.9	Users vs. non-users	462/27,383	0.92	[0.70–1.22]	0.6	Continuous	462/27,383	0.92	[0.78–1.09]	0.4
*Vitamin B2*	Q1	106/6831	1	(ref)	0.9	C1	406/23,998	1	(ref)	0.3	Q1	104/6832	1	(ref)	0.9
	Q2	120/6857	1.05	[0.80–1.37]		C2	22/1124	1.09	[0.71–1.67]		Q2	122/6853	1.08	[0.82–1.41]	
	Q3	111/6857	0.93	[0.70–1.23]		C3	22/1133	1.07	[0.70–1.65]		Q3	117/6849	1.00	[0.76–1.32]	
	Q4	125/6838	1.05	[0.79–1.41]		C4	12/1128	0.60	[0.34–1.07]		Q4	119/6849	1.01	[0.75–1.35]	
Niacin	Continuous	462/27,382	1.00	[0.98–1.01]	0.7	Users vs. non-users	462/27,382	0.86	[0.65–1.15]	0.3	Continuous	462/27,382	0.99	[0.98–1.01]	0.4
*Vitamin B3*	Q1	101/6841	1	(ref)	1.0	C1	410/24,046	1	(ref)	0.2	Q1	102/6835	1	(ref)	0.6
	Q2	120/6849	1.01	[0.81–1.38]		C2	18/1132	0.89	[0.55–1.42]		Q2	122/6848	1.06	[0.81–1.38]	
	Q3	123/6846	1.05	[0.80–1.38]		C3	22/1137	1.06	[0.69–1.63]		Q3	125/6846	1.04	[0.79–1.36]	
	Q4	118/6846	1.01	[0.76–1.34]		C4	12/1067	0.62	[0.35–1.11]		Q4	113/6853	0.94	[0.71–1.25]	
Pantothenic acid	Continuous	462/27,370	0.98	[0.91–1.07]	0.7	Users vs. non-users	462/27,370	0.82	[0.61–1.12]	0.2	Continuous	462/27,370	0.95	[0.89–1.01]	0.1
*Vitamin B5*	Q1	109/6827	1	(ref)	0.7	C1	416/24,277	1	(ref)	0.1	Q1	111/6820	1	(ref)	0.4
	Q2	122/6851	1.01	[0.77–1.32]		C2	14/1017	0.77	[0.45–1.32]		Q2	116/6856	0.94	[0.72–1.23]	
	Q3	109/6856	0.88	[0.66–1.16]		C3	22/976	1.24	[0.81–1.91]		Q3	125/6838	0.97	[0.74–1.28]	
	Q4	122/6836	0.99	[0.73–1.34]		C4	10/1100	0.50	[0.27–0.94]		Q4	110/6856	0.85	[0.63–1.15]	
Pyridoxine	Continuous	462/27,323	0.83	[0.66–1.05]	0.1	Users vs. non-users	462/27,323	0.84	[0.65–1.07]	0.2	Continuous	462/27,323	0.81	[0.71–0.94]	0.005
*Vitamin B6*	Q1	115/6808	1	(ref)	0.05	C1	386/22,302	1	(ref)	0.05	Q1	113/6808	1	(ref)	0.01
	Q2	123/6836	0.93	[0.72–1.21]		C2	33/1816	1.02	[0.71–1.45]		Q2	126/6827	0.96	[0.74–1.25]	
	Q3	122/6833	0.88	[0.67–1.16]		C3	25/1549	0.88	[0.58–1.31]		Q3	131/6822	0.96	[0.73–1.25]	
	Q4	102/6846	0.74	[0.55–0.99]		C4	18/1656	0.61	[0.38–0.98]		Q4	92/6866	0.67	[0.50–0.91]	
Folate	Continuous	462/27,386	1.00	[1.00–1.00]	0.4	Users vs. non-users	462/27,386	0.89	[0.67–1.18]	0.4	Continuous	462/27,386	1.00	[1.00–1.00]	0.2
*Vitamin B9*	Q1	116/6810	1	(ref)	0.8	C1	406/23,874	1	(ref)	0.2	Q1	117/6807	1	(ref)	0.6
	Q2	98/6879	0.74	[0.56–0.98]		C2	24/1035	1.30	[0.86–1.97]		Q2	100/6877	0.75	[0.57–0.99]	
	Q3	128/6841	0.95	[0.73–1.24]		C3	17/1404	0.66	[0.41–1.07]		Q3	127/6843	0.92	[0.71–1.21]	
	Q4	120/6856	0.88	[0.66–1.17]		C4	15/1073	0.80	[0.48–1.34]		Q4	118/6859	0.85	[0.64–1.13]	
Cobalamin	Continuous	462/27,378	1.01	[0.99–1.02]	0.5	Users vs. non-users	462/27,378	0.90	[0.64–1.28]	0.6	Continuous	462/27,378	1.00	[0.99–1.01]	1.0
*Vitamin B12*	Q1	97/6846	1	(ref)	0.8	C1	427/25,187	1	(ref)	0.4	Q1	96/6845	1	(ref)	0.8
	Q2	119/6851	1.08	[0.82–1.42]		C2	11/727	0.86	[0.47–1.57]		Q2	122/6848	1.11	[0.85–1.46]	
	Q3	123/6836	1.06	[0.81–1.41]		C3	17/729	1.25	[0.77–2.03]		Q3	121/6838	1.06	[0.80–1.40]	
	Q4	123/6845	1.05	[0.79–1.39]		C4	7/735	0.57	[0.27–1.19]		Q4	123/6847	1.06	[0.80–1.41]	

HR Hazard Ratio; CI, Confidence Interval; Ref, Reference; Q, quartile; C, class (C1 = non-users and C2 to C4 = tertiles of supplemental intake in users of the corresponding supplement) ^a^ Models were adjusted for age (time-scale), BMI (kg/m^2^, continuous), height (cm, continuous), physical activity (IPAQ categories: high, moderate, low, or missing), smoking status (never, former, current smokers), number of dietary records (continuous), alcohol intake (g/day, continuous), energy intake without alcohol (g/day, continuous), family history of cancer (yes/no), educational level (<high-school degree, <2 years after high-school degree, ≥2 years after high-school degree), number of biological children (continuous), menopausal status at baseline (pre-menopause, perimenopause, post-menopause), and baseline use of hormonal treatment for menopause (yes/no). For cobalamin, models were additionally adjusted for red meat consumption (g/day, continuous). ^b^ Cut-offs for quartiles of dietary intakes were 0.9/1.1/1.4 mg/day for thiamin, 1.4/1.7/2.1 mg/day for riboflavin, 14.8/18.3/22.4 mg/day for niacin, 4.4/5.2/6.2 mg/day for pantothenic acid, 1.4/1.7/2.0 mg/day for pyridoxine, 262.6/324.2/397.8 µg/day for folate, and 3.2/4.3/6.1 µg/day for cobalamin. Cut-offs for tertiles of supplemental intakes in users of the corresponding supplement were 0.1/0.3 mg/day for thiamin, 0.1/0.3 mg/day for riboflavin, 0.7/3.0 mg/day for niacin, 0.5/1.5 mg/day for pantothenic acid, 0.2/0.7 mg/day for pyridoxine, 16.4/49.3 µg/day for folate, and 0.1/0.4 µg/day for cobalamin. Cut-offs for quartiles of total intakes were 0.9/1.1/1.4 mg/day for thiamin, 1.4/1.7/2.1 mg/day for riboflavin, 15.0/18.6/22.9 mg/day for niacin, 4.4/5.3/6.4 mg/day for pantothenic acid, 1.4/1.7/2.2 mg/day for pyridoxine, 265.8/328.8/405.2 µg/day for folate, and 3.2/4.4/6.2 µg/day for cobalamin.

**Table 3:** Associations between B-vitamin supplement use and breast cancer risk, stratified by the median of alcohol intake, NutriNet-Santé Cohort, France, 2009–2016 ^a^.

	Cases/Non Cases	HR	95%CI	*P*	P-interaction ^b^
Thiamin					0.003
Alcohol intake < median					
Non-users of thiamin supplements	190/11,972	1	(ref)	0.01	
Users of thiamin supplements	15/1736	0.52	[0.30–0.87]		
Alcohol intake ≥ median					
Non-users of thiamin supplements	216/12,054	1	(ref)	0.09	
Users of thiamin supplements	41/1614	1.34	[0.96–1.88]		
Riboflavin					0.007
Alcohol intake < median					
Non-users of riboflavin supplements	188/11,970	1	(ref)	0.02	
Users of riboflavin supplements	16/1742	0.55	[0.33–0.93]		
Alcohol intake ≥ median					
Non-users of riboflavin supplements	218/12,028	1	(ref)	0.2	
Users of riboflavin supplements	40/1643	1.26	[0.90–1.76]		
Niacin					0.01
Alcohol intake < median					
Non-users of niacin supplements	189/12,002	1	(ref)	0.02	
Users of niacin supplements	15/1709	0.53	[0.31–0.90]		
Alcohol intake ≥ median					
Non-users of niacin supplements	221/12,044	1	(ref)	0.4	
Users of niacin supplements	37/1627	1.16	[0.82–1.64]		
Pantothenic acid					0.01
Alcohol intake < median					
Non-users of pantothenic acid supplements	193/12,134	1	(ref)	0.01	
Users of pantothenic acid supplements	12/1571	0.47	[0.26–0.84]		
Alcohol intake ≥ median					
Non-users of pantothenic acid supplements	223/12,143	1	(ref)	0.5	
Users of pantothenic acid supplements	34/1522	1.14	[0.79–1.64]		
Pyridoxine					0.02
Alcohol intake < median					
Non-users of pyridoxine supplements	179/11,123	1	(ref)	0.01	
Users of pyridoxine supplements	25/2558	0.59	[0.39–0.89]		
Alcohol intake ≥ median					
Non-users of pyridoxine supplements	207/11,179	1	(ref)	0.7	
Users of pyridoxine supplements	51/2463	1.07	[0.79–1.46]		
Folate					0.02
Alcohol intake < median					
Non-users of folate supplements	187/11,898	1	(ref)	0.04	
Users of folate supplements	18/1814	0.60	[0.37–0.98]		
Alcohol intake ≥ median					
Non-users of folate supplements	219/11,976	1	(ref)	0.4	
Users of folate supplements	38/1698	1.15	[0.81–1.62]		
Cobalamin					0.02
Alcohol intake < median					
Non-users of cobalamin supplements	196/12,586	1	(ref)	0.04	
Users of cobalamin supplements	9/1122	0.50	[0.26–0.97]		
Alcohol intake ≥ median					
Non-users of cobalamin supplements	231/12,601	1	(ref)	0.3	
Users of cobalamin supplements	26/1069	1.26	[0.84–1.90]		

HR Hazard Ratio; CI, Confidence Interval; Ref, Reference; ^a^ From multivariate Cox proportional hazards models. Median of daily alcohol intake was 3.6 g/day for women. Models were adjusted for age, BMI, height, physical activity, smoking status, number of dietary records, alcohol intake, energy intake without alcohol, family history of cancer, educational level, number of biological children, menopausal status at baseline, and hormonal treatment for menopause. For cobalamin, the models were also adjusted for red meat consumption (g/day, continuous); ^b^ Between B-vitamin supplement use and alcohol intake.

## Supplementary Materials

The following are available online at www.mdpi.com/2072-6643/9/5/488/s1, Table S1: Associations between dietary, supplemental, and total B vitamin intake and breast cancer risk, from age-adjusted Cox proportional hazards models ^a^, NutriNet-Santé Cohort, France, 2009–2016.
