# Supplementary material for: B-Vitamin Intake from Diet and Supplements and Breast Cancer Risk in Middle-Aged Women: Results from the Prospective NutriNet-Santé Cohort
Source: Nutrients. 2017 May 13;9(5):488. doi: 10.3390/nu9050488 (PMC5452218; doi:10.3390/nu9050488)
Supplement: Supplementary file 1 [file nutrients-09-00488-s001.pdf]

Supplemental Table 1. Associations between dietary, supplemental, and total B vitamin intake and breast cancer risk, from age-adjusted Cox proportional hazards models[a], NutriNet-Santé Cohort, France, 2009-2016.

		**Dietary intake**					**Supplemental intake**					**Total intake**			
		**Cases /non cases**	**HR**	**95%CI**	***P*-trend**		**Cases /non cases**	**HR**	**95%CI**	***P*-trend**		**Cases /non cases**	**HR**	**95%CI**	***P*-trend**
Thiamin	Continuous	462/27,376	0.90	[0.70–1.15]	0.4	Users vs non-users	56/3350	0.93	[0.71–1.23]	0.6	Continuous	462/27,376	0.83	[0.67–1.03]	0.09
Vitamin B1	Q1[b]	121/6820	1	(ref)	0.3	C1	406/24,026	1	(ref)	0.2	Q1	120/6817	1	(ref)	0.2
	Q2	113/6857	0.88	[0.68–1.14]		C2	26/1125	1.32	[0.89–1.97]		Q2	110/6853	0.87	[0.67–1.12]	
	Q3	125/6848	0.97	[0.76–1.25]		C3	18/1099	0.89	[0.56–1.43]		Q3	134/6844	1.05	[0.82–1.34]	
	Q4	103/6851	0.83	[0.64–1.08]		C4	12/1126	0.59	[0.33–1.05]		Q4	98/6862	0.79	[0.60–1.03]	
Riboflavin	Continuous	462/27,383	1.04	[0.87–1.25]	0.6	Users vs non-users	56/3385	0.93	[0.70–1.22]	0.6	Continuous	462/27,383	0.96	[0.83–1.10]	0.5
Vitamin B2	Q1	106/6831	1	(ref)	0.6	C1	406/23,998	1	(ref)	0.3	Q1	104/6832	1	(ref)	0.8
	Q2	120/6857	1.07	[0.83–1.39]		C2	22/1124	1.10	[0.71–1.68]		Q2	122/6853	1.11	[0.85–1.44]	
	Q3	111/6857	0.97	[0.74–1.26]		C3	22/1133	1.07	[0.70–1.65]		Q3	117/6849	1.04	[0.80–1.35]	
	Q4	125/6838	1.11	[0.85–1.43]		C4	12/1128	0.60	[0.34–1.07]		Q4	119/6849	1.06	[0.82–1.38]	
Niacin	Continuous	462/27,382	1.00	[0.99–1.02]	0.9	Users vs non-users	52/3336	0.86	[0.65–1.15]	0.3	Continuous	462/27,382	1.00	[0.98–1.01]	0.7
Vitamin B3	Q1	101/6841	1	(ref)	0.6	C1	410/24,046	1	(ref)	0.2	Q1	102/6835	1	(ref)	0.9
	Q2	120/6849	1.10	[0.85–1.44]		C2	18/1132	0.89	[0.56–1.43]		Q2	122/6848	1.11	[0.85–1.44]	
	Q3	123/6846	1.12	[0.86–1.45]		C3	22/1137	1.06	[0.69–1.63]		Q3	125/6846	1.11	[0.86–1.45]	
	Q4	118/6846	1.09	[0.83–1.42]		C4	12/1067	0.62	[0.35–1.10]		Q4	113/6853	1.02	[0.78–1.34]	
Pantothenic acid	Continuous	462/27,370	1.00	[0.94–1.07]	0.7	Users vs non-users	46/3093	0.83	[0.61–1.12]	0.2	Continuous	462/27,370	0.97	[0.92–1.02]	0.3
Vitamin B5	Q1	109/6827	1	(ref)	0.9	C1	416/24,277	1	(ref)	0.1	Q1	111/6820	1	(ref)	0.7
	Q2	122/6851	1.05	[0.81–1.36]		C2	14/1017	0.79	[0.46–1.33]		Q2	116/6856	0.99	[0.76–1.29]	
	Q3	109/6856	0.93	[0.71–1.21]		C3	22/976	1.25	[0.81–1.91]		Q3	125/6838	1.05	[0.81–1.35]	
	Q4	122/6836	1.06	[0.82–1.38]		C4	10/1100	0.50	[0.27–0.93]		Q4	110/6856	0.94	[0.72–1.22]	
Pyridoxine	Continuous	462/27,323	0.90	[0.74–1.09]	0.1	Users vs non-users	76/5021	0.83	[0.65–1.07]	0.1	Continuous	462/27,323	0.85	[0.75–0.96]	0.01
Vitamin B6	Q1	115/6808	1	(ref)	0.2	C1	386/22,302	1	(ref)	0.05	Q1	113/6808	1	(ref)	0.08
	Q2	123/6836	0.99	[0.77–1.28]		C2	33/1816	1.01	[0.71–1.44]		Q2	126/6827	1.02	[0.79–1.32]	
	Q3	122/6833	0.97	[0.75–1.25]		C3	25/1549	0.88	[0.59–1.31]		Q3	131/6822	1.06	[0.82–1.36]	
	Q4	102/6846	0.83	[0.63–1.08]		C4	18/1656	0.60	[0.38–0.96]		Q4	92/6866	0.75	[0.57–0.99]	
Folate	Continuous	462/27,386	1.00	[1.00–1.00]	0.5	Users vs non-users	56/3512	0.89	[0.67–1.17]	0.4	Continuous	462/27,386	1.00	[1.00–1.00]	0.3
Vitamin B9	Q1	116/6810	1	(ref)	0.9	C1	406/23,874	1	(ref)	0.2	Q1	117/6807	1	(ref)	0.7
	Q2	98/6879	0.77	[0.59–1.01]		C2	24/1035	1.31	[0.87–1.98]		Q2	100/6877	0.78	[0.60–1.02]	

	Q3	128/6841	0.98	[0.76–1.26]		C3	17/1404	0.67	[0.41–1.08]		Q3	127/6843	0.96	[0.75–1.24]	
	Q4	120/6856	0.91	[0.71–1.18]		C4	15/1073	0.78	[0.47–1.30]		Q4	118/6859	0.89	[0.69–1.15]	
Cobalamin	Continuous	462/27,378	1.01	[0.99–1.03]	0.3	Users vs non-users	35/2191	0.91	[0.65–1.29]	0.6	Continuous	462/27378	1.00	[0.99–1.01]	0.8
Vitamin B12	Q1	97/6846	1	(ref)	0.4	C1	427/25,187	1	(ref)	0.4	Q1	96/6845	1	(ref)	0.4
	Q2	119/6851	1.14	[0.87–1.49]		C2	11/727	0.87	[0.48–1.58]		Q2	122/6848	1.18	[0.90–1.54]	
	Q3	123/6836	1.15	[0.88–1.50]		C3	17/729	1.28	[0.79–2.07]		Q3	121/6838	1.14	[0.87–1.49]	
	Q4	123/6845	1.15	[0.88–1.49]		C4	7/735	0.57	[0.27–1.20]		Q4	123/6847	1.16	[0.89–1.52]	

Q, quartile; C, class (C1 = non-users and C2 to C4 = tertiles of supplemental intake in users of the corresponding supplement) [a] Models were only age-adjusted (time-scale in Cox models).[b] Cut-offs for quartiles of dietary intakes were 0.9/1.1/1.4 mg/d for thiamin, 1.4/1.7/2.1 mg/d for riboflavin, 14.8/18.3/22.4 mg/d for niacin, 4.4/5.2/6.2 mg/d for pantothenic acid, 1.4/1.7/2.0 mg/d for pyridoxine, 262.6/324.2/397.8 μg/d for folate, and 3.2/4.3/6.1 μg/d for cobalamin. Cut-offs for tertiles of supplemental intakes in users of the corresponding supplement were 0.1/0.3 mg/d for thiamin, 0.1/0.3 mg/d for riboflavin, 0.7/3.0 mg/d for niacin, 0.5/1.5 mg/d for pantothenic acid, 0.2/0.7 mg/d for pyridoxine, 16.4/49.3 μg/d for folate, and 0.1/0.4 μg/d for cobalamin. Cut-offs for quartiles of total intakes were 0.9/1.1/1.4 mg/d for thiamin, 1.4/1.7/2.1 mg/d for riboflavin, 15.0/18.6/22.9 mg/d for niacin, 4.4/5.3/6.4 mg/d for pantothenic acid, 1.4/1.7/2.2 mg/d for pyridoxine, 265.8/328.8/405.2 μg/d for folate, and 3.2/4.4/6.2 μg/d for cobalamin.
